# The Neural Dynamics of Fronto-Parietal Networks in Childhood Revealed using Magnetoencephalography

**DOI:** 10.1093/cercor/bhu271

**Published:** 2014-11-19

**Authors:** Duncan E. Astle, Henry Luckhoo, Mark Woolrich, Bo-Cheng Kuo, Anna C. Nobre, Gaia Scerif

**Affiliations:** 1 MRC Cognition and Brain Sciences Unit, Cambridge, UK; 2 Oxford Centre for Human Brain Activity (OHBA), University of Oxford, Oxford, UK; 3 Department of Experimental Psychology, University of Oxford, Oxford, UK; 4 Department of Psychology, National Taiwan University, Taipei, Taiwan

**Keywords:** cognitive control, cognitive development, development, executive control, magnetoencephalography

## Abstract

Our ability to hold information in mind is limited, requires a high degree of cognitive control, and is necessary for many subsequent cognitive processes. Children, in particular, are highly variable in how, trial-by-trial, they manage to recruit cognitive control in service of memory. Fronto-parietal networks, typically recruited under conditions where this cognitive control is needed, undergo protracted development. We explored, for the first time, whether dynamic changes in fronto-parietal activity could account for children's variability in tests of visual short-term memory (VSTM). We recorded oscillatory brain activity using magnetoencephalography (MEG) as 9- to 12-year-old children and adults performed a VSTM task. We combined temporal independent component analysis (ICA) with general linear modeling to test whether the strength of fronto-parietal activity correlated with VSTM performance on a trial-by-trial basis. In children, but not adults, slow frequency theta (4–7 Hz) activity within a right lateralized fronto-parietal network in anticipation of the memoranda predicted the accuracy with which those memory items were subsequently retrieved. These findings suggest that inconsistent use of anticipatory control mechanism contributes significantly to trial-to-trial variability in VSTM maintenance performance.

## Introduction

Many cognitive functions rely on our ability to hold in mind small amounts of information for brief periods of time. Importantly, the capacity of this short-term store is variable across individuals and increases gradually with development (Astle et al. 2012; Astle and Scerif 2012; Finn et al. 2010; Ciesielski et al. 2010; Sander et al. 2012). Variability in maintenance abilities from child to child predicts the amount of progress a child makes in classroom learning (Bull et al. 2008; Gathercole and Pickering 2000; Gathercole et al. 2005). However, for any given child, short-term memory performance is highly variable; in typically developing children, visual short-term memory (VSTM) and working memory performance is characterized by inconsistency. The neural correlates of memory successes and failures have been studied extensively in adults, using the subsequent memory paradigm (e.g., Brewer et al. 1998; Wagner et al. 1998). However, this trial-to-trial variability is less well studied in children, even though it is a core feature of immature memory performance in childhood. Here, we are interested in the dynamic neural mechanisms that underpin trial-to-trial variability in short-term memory in a group of children. In our experimental design, we equated the performance of children and adults. This enabled us to focus on the role of fluctuations in the activity of particular functional networks in this performance variability, within each group.

There is extensive overlap in the mechanisms and neural correlates of VSTM and attentional control (Awh and Jonides 2001; Awh et al. 2006; Gazzaley and Nobre 2012; Astle et al. 2012). Attentional control appears to optimize visual perception by enhancing neural signals carrying information most relevant to the task at hand and/or suppressing those carrying task-irrelevant information (Desimone and Duncan 1995). Similar control mechanisms may also underpin VSTM maintenance (e.g., Sakai and Passingham 2003; Gazzaley and Nobre 2012). When maintaining information in VSTM, domain-general areas in frontal and parietal cortices (e.g., prefrontal cortex, frontal eye fields and the intra-parietal sulcus) have direct or indirect input into the domain-specific sensory areas that originally processed the material and make these representations durable over a short maintenance delay (e.g., Sakai and Passingham 2003; Rutman et al. 2010; Mayer et al. 2007). In adulthood, variability in the recruitment of these frontal and parietal areas as subjects process memoranda correlates significantly with each subject's VSTM capacity (Linke et al. 2012). One possibility is that these areas play an important role in regulating the efficient sensory processing and encoding of the memoranda, and this is why their recruitment appears to be so important for good VSTM performance. It is unclear whether or not we would expect these mechanisms to explain trial-to-trial memory failures and successes in both children and adults, or whether the mechanisms underpinning this kind of variability in childhood would be qualitatively different to those important in the adult end state.

Here, we aimed to assess whether the recruitment of fronto-parietal cortices may explain some of the variability in memory performance in childhood. Recent research has shown that functional networks purported to subserve lower-level sensory and motor systems are in place relatively early on in development (de Bie et al. 2012). In contrast, connections between the lateral frontal cortices and other cortical and subcortical areas show a particularly protracted development (Giedd et al. 1999). This protracted development, particularly in the case of lateral fronto-parietal networks, mirrors the more gradual development of cognitive control, attention and VSTM (e.g., Huizinga et al. 2006). Even by late childhood, networks associated with these higher-order cognitive functions remain immature, namely the fronto-parietal networks (de Bie et al. 2012). This includes connections between dorso-lateral prefrontal cortex and intra-parietal lobules and sulci (e.g., Fair et al. 2007). Instead, these networks are characterized at rest by fragmentation relative to the equivalent adult networks (de Bie et al. 2012). Furthermore, failures of these networks are implicated in a large number of neurodevelopmental disorders (e.g., Fair et al. 2010). For example, children with Tourette's Syndrome have less “mature” fronto-parietal networks, relative to age-matched controls (Church et al. 2009). This immaturity was characterized by atypical connections between regions including the dorso-lateral prefrontal cortices, intra-parietal lobule and sulcus, and mid-cingulate gyrus.

Despite previous studies exploring the relative immaturity of these networks, either in terms of their structural connections, or their resting-state connectivity, there has been little attempt to explore how dynamic changes in the levels of activity or connectivity within these networks underpin cognitive performance in childhood. Here, we focus on how endogenous fluctuations in the activity patterns of these networks are linked to trial-to-trial variability in VSTM performance. In this study, we focus on a group of children aged 9–12 years. Unlike younger children, children of this age are capable of achieving relatively complicated adult-like maintenance in memory (e.g., Astle et al. 2012; Shimi, Nobre, Astle and Scerif 2014; Shimi, Kuo, Astle, Nobre and Scerif 2014). However, as described earlier, those fronto-parietal connections purported to underpin higher-order cognition remain relatively underdeveloped. As a result, they provide a particularly interesting comparison group with adults, because they are clearly capable of deploying some aspects of cognitive control, but the reliability with which these fronto-parietal networks can be recruited on a trial-to-trial basis may differ relative to adults.

### Using Magnetoencephalography to Study Functional Connectivity

It is becoming increasingly apparent that investigating dynamic functional connectivity is fundamental to understanding typical brain function in adulthood. This is particularly the case where one is interested in control functions, as these necessarily depend upon the strength of the relationship between those areas that have a controlling influence (e.g., dorso-lateral prefrontal cortex [DLPFC]) and those areas upon which the control is exerted (e.g., sensory areas). Functional networks are likely to be constituted by the spontaneous synchronization of neural oscillations across spatially distinct neuronal populations (Uhlhaas and Singer 2010; Buzsaki 2011). A number of recent studies have attempted to identify different functional networks using magnetic resonance imaging (MRI) of both resting and active brain states (e.g., Dosenbach et al. 2008; Dosenbach et al. 2007; Fair et al. 2008; Fair et al. 2007; Power et al. 2010). However, an important limitation of this approach is that measures of network activity obtained through MRI are over a relatively long timescale. More recently, magnetoencephalography (MEG) has been used to identify functional networks from data acquired from the adult brain (Brookes et al. 2011). Whereas BOLD provides an indirect measure of neural activity related to hemodynamic changes, MEG provides a direct “electrophysiological measure” of the activity underpinning functional connectivity. In addition, MEG signals enable the identification of different spatially distributed networks that are distinct in their “temporal” structure. For instance, Brookes et al. (2011) used a temporal independent component analysis (ICA) of the low-frequency band-limited power fluctuations in source space MEG data to identify 8 distinct functional networks, including the default-mode network and lateral fronto-parietal networks. The increased temporal precision of MEG may confer an important advantage in the study of functional networks: The accurate timing of when a particular network is recruited for a particular cognitive operation will in turn enable the more precise allocation of cognitive function to that particular network. Harnessing this temporal precision was a central aim of the current study; critically, we were able to distinguish anticipatory and poststimulus processing.

We used MEG data to identify the cortical networks recruited by a group of typically developing children as they maintained items in VSTM. We focused on networks involving lateral prefrontal cortices, because biasing signals from these areas are thought to play a critical role in modifying neural activity in other cortical areas. These biasing mechanisms can be deployed both in anticipation of stimulus onset and during maintenance, both of which may be particularly important for VSTM performance (Gazzaley and Nobre 2012). A unique strength of this approach is that not only were we able to identify lateral fronto-parietal networks, but we could explore their role in VSTM performance at particular moments in time. In this study, we draw a particular distinction between pre- and post-stimulus activity. In the process of addressing this particular question, we also wanted to establish a methodology to probe the electrodynamics of functional connectivity during childhood. We also collected data from a group of young adults performing a task matched for difficulty with that performed by the children. This comparison group enabled us to validate our ICA result against existing uses of this approach with adults (e.g., Luckhoo et al. 2012), as well as explore the similarity in dynamic fronto-parietal activity across these 2 groups.

## Methods

### Participants

Thirteen children, aged 9–12 years (*N* = 13, mean age 11.15, SD 0.87, 4 were male), participated in the experiment, although 1 child could not be used for any analyses due to a high number of eye movement artifacts. As outlined in Introduction, this age range was chosen because these children have yet to undergo fully the developmental maturation fronto-parietal functional connectivity. Each child provided verbal assent, and his or her parent provided written informed consent. We also collected a comparison data set from a group of adults (*N* = 10, mean age 27, SD 4.85, 6 were male), who were paid £30 for their participation. All subjects were right-handed. The University of Oxford Central University Research Ethics Committee provided permission for the study.

### Behavioral Task

**Figure 1.**
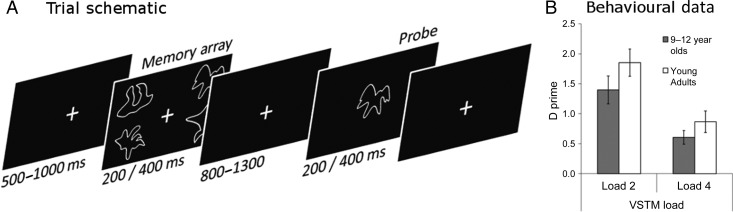
(*A*) Trial schematic showing an example of a VSTM Load-4 target present trial, with the timings for both children and adults. (*B*) The behavioral data, shown as *d*′ values for the 2 groups, across the 2 VSTM load conditions. The error bars refer to the standard error of the mean.

Subjects were presented with arrays of to-be-remembered shapes, in set sizes of either 2 or 4 items. Subjects retained these for a brief retention delay (∼1 s), after which a single probe shape appeared. Subjects reported whether the probe was present or absent from the previous array. A trial-order schematic is presented in Figure 1*A*. We also included a number of visual search trials, in which subjects were first shown the probe shape and had to perform a covert visual search for it in a subsequent array of 2 or 4 items (i.e., the reverse of the VSTM trials). These trials were included in the ICA, because the additional data points that they provide improve the characterization of the networks, but we did not submit these trials to the subsequent general linear model (GLM) because these trials were not relevant to the experimental question being addressed.

### Task Design

Subjects were seated 100 cm from the screen on which the stimuli were projected. Subjects were first presented with a fixation cross for 500–1000 ms, after which a memory array was presented. The memory array was then presented for 200 ms (for adults) or 400 ms (for children), followed by a maintenance variable interval lasting between 800 and 1300 ms. At the end of this interval, subjects were presented with a single probe shape at fixation for 200 ms (for adults) or 400 ms (for children). They responded with their right hand, using their index and middle fingers to respond “present” or “absent,” respectively. Present and absent trials were equally likely. For adults, the next trial started after a delay of 2500–3000 ms regardless of whether they responded. For children, trials were organized with blocks of 20 trials, after which a time-limited break was given. After 5 of these blocks, the child was given a longer self-timed break. Each child performed 4 of these 100 trial runs, with each taking ∼11 min. The adults performed 4 slightly longer blocks of 15 min and timed their own within-run breaks. We used slightly longer presentation durations for the children relative to the adults, because we did not want children's performance to be limited by slow encoding (e.g., Cowan et al. 2011). While this ensures that children and adults are more closely matched in behavioral terms and a sufficient number of successful trials in the children's data, it does limit the extent to which we can compare directly the neural effects in children and adults, and these are therefore analyzed separately.

### Stimuli

A black background was used throughout. The stimuli used for all the trials were drawn from a set of novel meaningless closed shape contours previously developed by Endo and colleagues (Endo et al. 2003), which are difficult to verbalize. All of the shapes were white. The memory arrays were composed of 2 (50%) or 4 (50%) shapes randomly selected from a subset of 8 items. Each stimulus was 1.72° visual angle in size and was positioned randomly in 1 of the 4 possible peripheral locations of an invisible 2 × 2 matrix. Stimulus locations were centered at 2.58° eccentricity and 2° elevation. Two-item arrays contained stimuli presented along a diagonal (top left and bottom right or vice versa).

### Behavioral Analyses

Subjects' ability to detect the presence of the probe stimuli in the preceding memory array was determined using *d*′ scores (Green and Swets 1966). In this case, *d*′ scores correspond to subjects' sensitivity to detect the presence of the probe item in the VSTM array. It is calculated by comparing the normalized proportion for correct hits to the normalized proportion of false alarms. We submitted the *d*′ scores to a two-way ANOVA, with memory load (set size 2 versus 4) as a within-subjects factor, and group as a between-subjects factor (children versus adults).

### MEG Data Acquisition

We acquired the data using a 306-channel VectorView system (Elekta Neuromag), with 1 magnetometer and 2 orthogonal planar gradiometers at each of the 102 locations. Subjects were seated in a magnetically shielded room. The data were sampled at 1000 Hz, and band pass filtered at 0.03–330 Hz. For the adults, we monitored head position at the start and at the end of each of the 4 runs using 4 head position indicator (HPI) coils fixed to the head. For the children, we recorded continuous head position throughout each run, such that we could correct for any head movements during our analyses. For each subject, we used a Polhemus Isotrak II to digitize the locations of fiducial markers (the nasion, left and right pre-auricular points), the HPI coils and a number of additional head points (these were subsequently used to aid our co-registration). Vertical and horizontal electro-oculograms were recorded and were subsequently used to remove trials contaminated by blinks or eye-movements.

### MEG Data Preprocessing

External noise was removed from the MEG data using the signal-space separation method, and adjustments in head position across runs were compensated for using the MaxMove software, both implemented in MaxFilter version 2.1 (Elekta Neuromag). The MaxFilter software works by mathematically transforming the data to a set of virtual sensors. This is possible because the software has very accurate location measurements of both the MEG sensor array and the subject's head position. Transforming the data into virtual sensors enables the researcher to set a standard reference frame and thus combine easily across the recordings. For the children, we also used this software to correct for head movements within each session. There has been recent interest in the role that head movement artifact may play in patterns of neural activity in pediatric samples (Power et al. 2012). Because of our continuous recording of each child's head position, we could check at each sample of the MEG recording that the child's head was accurately transformed to the standard reference frame – thereby controlling for any within-session head movements. Transforming the data to a set of virtual sensors also allows for the removal of noise emanating from outside the scanner. This signal-space separation method acts to suppress any activity that does not stem from virtual channels within the helmet.

The data were subsequently down-sampled to 250 Hz using SPM8 (http://www.fil.ion.ucl.ac.uk/spm/), and epoched from −400 to 800 ms relative to the onset of the memory array. Trials contaminated by eye-blinks or movements were removed manually by inspecting the amplitude time courses of the raw eye-channel data. We also removed outlier trials with abnormally high variance using the FieldTrip visual artifact rejection routines (Oostenveld et al. 2011). This tool works by calculating the amplitude variance in the raw signal across the entire window of each trial. Trials with an overall variance that was above 2 standard deviations beyond what was typical for that subject were removed.

### MEG Source Reconstruction

We co-registered each subject's MEG data to a standard MNI template using the digitized scalp locations and fiducials via an iterative closest point algorithm using SPM8 (http://www.fil.ion.ucl.ac.uk/spm/). Prior to beamforming, we bandpass filtered the data such that we focused only on the slower frequencies (theta: 4–7 Hz); previous work on short-term memory has focused on these slower frequencies (Axmacher et al. 2010; Liebe et al. 2012). Furthermore, previous work has shown that these slower frequencies are better for exploring functional connections with MEG and increase discrimination between spurious and genuine connectivity (Luckhoo et al. 2012). For each subject, we estimated the source space activity at every vertex of a 7-mm grid covering the entire brain, using a linearly constrained minimum variance beamformer (Van Veen et al. 1997). The beamformer combined information from both the magnetometers and planar gradiometers while taking into account the reduced dimensionality of the data introduced by the signal-space separation algorithm (Woolrich et al. 2011). Beamforming constructs a spatial filter, which is applied to the sensor data to reconstruct the signal at each grid point, with the aim of achieving unit bandpass response at the grid point while minimizing the variance passed from all other locations. In short, the data at the source location of interest are given by multiplying the beamformer weights vector by the original sensor data. The process can be repeated across all grid locations to achieve a whole brain source reconstruction. The data covariance matrix was also regularized according to Creg = *C* + μI, where μ equals 4 times the minimum eigenvalue of the unregularized data covariance matrix, *C*.

### Independent Components Analysis

The oscillatory amplitude envelopes of the reconstructed time series were estimated via computation of the absolute value of the analytic signal, which was found using a Hilbert Transform, yielding an estimate of instantaneous signal amplitude at each voxel. The envelope time series for every voxel was effectively low-pass filtered by dividing each envelope time course into 0.1-s windows and averaging within those windows, focusing on low-frequency power fluctuations that are thought to be direct manifestations of electrophysiological functional connectivity (Brookes et al. 2011).

Once we had down-sampled amplitude envelopes for all source space voxels and all subjects, we temporally concatenated them across trials and subjects. Temporal ICA was then performed using fastICA, in which the data were reduced to 20 dimensions using a Principle Component Analysis (Luckhoo et al. 2012). This results in 20 temporally independent time series, which were each converted into correlation spatial maps by estimating the Pearson correlation coefficient between each independent time course and the down-sampled amplitude envelope time course, concatenated across subjects, associated with each voxel. That is, the maps show the extent to which each voxel correlates with a particular component, and therefore, each map provides the spatial distribution of each component identified by the ICA.

Alongside the ICA, a GLM (Woolrich et al. 2009) was implemented. The GLM assumes that a signal can be modeled as a linear combination of known regressors and a normally distributed error. This was done by splitting the concatenated ICs back into individual subject time courses, and then applying a mass univariate linear regression, or GLM approach. The GLM design matrix has a regressor for each condition of interest, with ones indicating those trials that belong to the condition in question, and zeros otherwise. We used 2 contrasts for both the children and adults. The first of these used only correct trails and identified the main effect of load (VSTM load 4 greater than VSTM load 2). For the second of these, we coded the accuracy on each trial (using responses to the probe shape at the end of the trial) and then used this to create a contrast that identified accuracy effects (VSTM load 4 trials upon which the subject was subsequently correct versus VSTM load 4 trials upon which the subject was subsequently incorrect). We used 2 separate GLMs for each contrast, the first applied to the prestimulus interval (collapsing across the time points in the prestimulus period) and the second applied to the poststimulus interval (collapsing across the time points in the poststimulus period). We did this because some components might distinguish significantly the trials prior to the onset of the stimuli. For instance, prestimulus theta fluctuations may distinguish trials that are subsequently remembered versus forgotten. In contrast, other components may distinguish trials during the maintenance period; for instance, poststimulus theta fluctuations may distinguish Load 2 versus Load 4 trials.

When applying the GLM to the independent time courses, a multi-step-up test was used to correct for multiple comparisons over components (Nichols and Hayasaka 2003). The ICs were ranked by the significance of the effect of each regressor, from the least to the most significant. In Step 1, the least significant component was tested for significance against a threshold (*P* = 0.05). If it proved to be non-significant, the next least significant *P*-value was selected (Step 2) and multiplied by a scaling factor equal to the step number. This continues until 1 of the components survives the correction, and all remaining (more significant) components were then classed as above threshold. A step-up correction was used as it is equivalent to the FDR corrections applied to traditional GLM analyses (Nichols and Hayasaka 2003). In our results, we focused on the fronto-parietal networks that our ICA produced. We plotted the activity of the right lateralized fronto-parietal IC at each of our samples (12 per trial), allowing us to compare the IC across our conditions in real time.

## Results

### Behavioral Results

There was a significant main effect of memory load [*F*_1,23_ = 64.172, *P* < 0.001], with performance being significantly better on Load 2 trials [*d*′ = 1.60] than on Load 4 trials [*d*′ = 0.72]. However, there was no significant effect of group [*F*_1,20_ = 2.037, *P* = 0.17], and there was no significant interaction between memory load and group [*F*_1,23_ = 0.770, *P* = 0.391]. Given that we subsequently used the Load 4 trials in our GLM looking at accuracy, we compared directly the accuracy of the 2 groups on this trial type, and they did not differ significantly [*t*_(20)_ = 1.261, *P* = 0.22]. The behavioral data for both groups can be seen in Figure 1*B*.

### Results of the ICA/GLM Analysis

**Figure 2.**
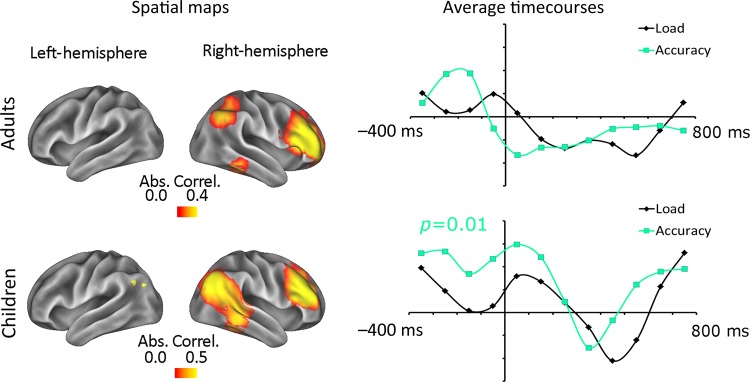
The right lateralized fronto-parietal identified using the ICA and GLM in both adults (upper panel) and children (lower panel). The left hand images show the spatial extent of the component networks (in terms of the absolute Pearson Correlation values between each brain location and this component); the right hand images show the time course of the GLM contrast for these networks. The black line reflects the effect of VSTM load, and the cyan line reflects the effect of subsequent accuracy.

Our main experimental question was whether dynamic changes in the state of activity within fronto-parietal areas contribute to trial-to-trial variability of children's VSTM performance. The ICA successfully identified a right-lateralized fronto-parietal network, which looked very similar across the 2 groups of subjects. In our data set, the left-hemisphere counterpart could not be identified in either group. We used the GLM to test whether activity in the right-hemisphere fronto-parietal network was significantly modulated by either the load or the accuracy contrast, in either the pre- or post-stimulus period, for each group. The spatial map and time course of these effects can be seen in Figure 2.

#### General Linear Model Result

In both children and adults, the IC comprised the right lateral prefrontal cortex and the right lateral parietal cortex. In the children, the activity of this component was not significantly modulated by trial-to-trial changes in VSTM load, either prior to the onset of the memoranda (*P*_corrected_ = 0.27) or following their onset (*P*_corrected_ = 0.38). The activity of this component was however modulated by trial-to-trial changes in the child's accuracy, but only during in the prestimulus period (*P*_corrected_ = 0.01), and not in the poststimulus period (*P*_corrected_ = 0.13). In the adults, the activity of this component was not modulated significantly by memory load (prestimulus *P*_corrected_ = 0.18; poststimulus *P*_corrected_ = 0.13) or accuracy (prestimulus *P*_corrected_ = 0.21; poststimulus *P*_corrected_ = 0.08).

#### Follow-up Analysis

The prestimulus result in the children was particularly interesting; it implied that the state of activation of the component network prior to the onset of the memoranda was important in predicting whether or not those memoranda would be successfully retained. One possibility is that this network has an important regulatory role in shaping the sensory processing of the memoranda, as suggested is the case in adults (e.g., Linke et al. 2012). We tested this with a follow-up analysis.

**Figure 3.**
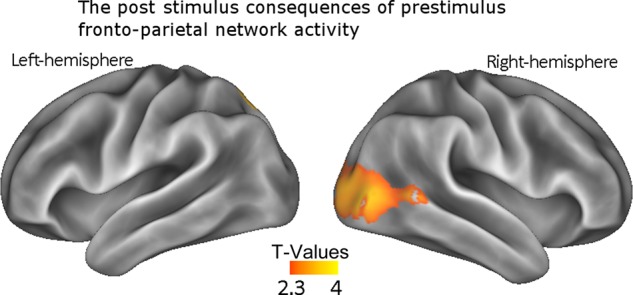
The supplementary analysis performed on the children's data. Activity in the right fronto-parietal network was used as a continuous trial-wise regressor, the effect of which upon the subsequent processing of the memoranda can be seen here.

We used the IC to create a spatial mask that comprised the right-hemisphere fronto-parietal network (all areas with a Pearson correlation of greater than 0.5). We used this mask, in combination with our down-sampled Hilbert envelope data, to identify the mean prestimulus activity within this network on each trial (collapsing across those time points in the prestimulus period). This resulted in a value for each trial, corresponding to the prestimulus activity in the right-lateralized fronto-parietal network. These values were then used as a continuous trial-wise regressor on the first 200 ms of the poststimulus period; this is intended to test whether the state of lateral prefrontal and parietal activations, which were influenced prior to the onset of the stimuli, would influence the early visual processing of those memoranda. This analysis essentially tests for temporally lagged connectivity. It probes whether prestimulus activity in the fronto-parietal network can influence poststimulus processing and, if so, it identifies where in the brain this influence is apparent. We only used Load-4 trials to make this comparison. In this way, it was possible to compare failed versus successful trials that were perceptually identical, and thus, any influence on visual processing must reflect an endogenous mechanism rather than differences in sensory input. This analysis revealed activity in the early and lateral visual areas (peak MNI: *x* = 16, *y* =− 94, *z* =− 10), ipsilateral to the fronto-parietal network, which can be seen in Figure 3. This also revealed a small portion of cerebellum (peak MNI: *x* = 20, *y* =− 80, *z* =− 32). In short, the anticipatory fronto-parietal activity is not only predictive of subsequent VSTM performance but also of subsequent levels of sensory activity when processing the memoranda. On trials when prestimulus levels of fronto-parietal activity are high, there are greater levels of visual activity following the onset of the memoranda, as well as greater memory performance at the end of trial.

**Figure 4.**
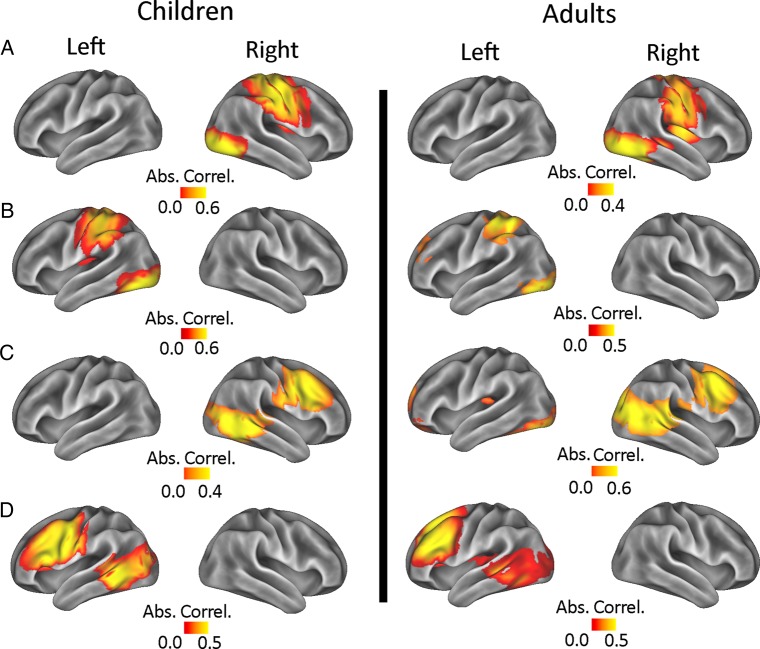
A set of additional components produced by the temporal ICA that were present in both adults and children. (*A* and *B*) Lateralized motor-visual networks that were right and left lateralized, respectively. (*C* and *D*) Lateralized frontal-visual networks that were right and left lateralized, respectively. As in Figure 2, the spatial maps are produced by calculating the absolute Pearson Correlation values between each brain location and each component.

In addition to the right fronto-parietal network, which was of direct interest to our experimental question, we were also able to identify other consistent networks. While they are not of direct interest here, the temporal ICA also produced a number of other components in both independent data sets (children and adults). These can be seen in Figure 4. They include a right-hemisphere component that comprises lateral visual areas and the motor cortex (A), and a corresponding component in the left hemisphere (B). We also observed a component that comprised the lateral frontal cortex and lateral visual areas in both groups, for both the right (C) and left hemisphere (D). Though the purpose of this study is not to give an exhaustive list of all of the ICs that can be observed by applying a temporal ICA to the time courses of oscillatory data (see Brookes et al. 2011), it is noteworthy that these are robustly present in children as well as in adults.

## Discussion

In the present study, we demonstrated a significant link between activity in the fronto-parietal network and the marked variability that children display in VSTM performance. Our results go further, in suggesting that dynamic fluctuations of activity in the fronto-parietal network regulate the state of visual excitability in preparation for memory encoding. Changes in spontaneous top-down attention modulation therefore may contribute significantly to variability in children's VSTM performance because they regulate the sensory processing of the memoranda. We explored synchronized power relationships across different cortical areas, using an ICA as a blind source separation technique to decompose the mixture of low-frequency signals (4–7 Hz) into distinct components (see also Luckhoo et al. 2012). By combining these results with a GLM, we were able to explore the relationship between dynamic changes in activity within this network and trial-to-trial VSTM performance variability in children and adults. We were consequently able demonstrate the down-stream consequences of activity within this network on activity within visual processing areas.

### Prestimulus Fronto-Parietal Activity and VSTM

We used an ICA to isolate a right-hemisphere network including the lateral frontal and posterior parietal cortices. This component relates closely to the fronto-parietal networks described previously (e.g., Fair et al. 2007; Duncan 2010; de Bie et al. 2012). In children, but not in adults, activity in this network significantly predicted trial-to-trial variability in VSTM accuracy. By splitting the GLM into a pre- and post-stimulus analysis, we were able to show that for the children this network was significantly modulated in anticipation of the memoranda. Thus, the MEG reveals a critical characteristic of how this network may influence children's VSTM performance – its activity level in anticipation of the memoranda is critical for successful memory performance. That this network has an anticipatory role would suggest that it plays an attentional role, rather than mainly a role in the active maintenance of the memoranda.

A consistent result from elsewhere is that this network is recruited under conditions requiring top-down control. This flexible top-down system can provide the necessary organizational input to lower-level domain-specific sensory systems, according to fluctuating task goals (Duncan 2010; Gazzaley and Nobre 2012). We suggest that the anticipatory activity of this network is critical for VSTM because it acts to regulate the subsequent sensory processing of the memoranda. Accordingly, we demonstrated that prestimulus activity in this network modulated subsequent visual processing, consistent with this network acting to regulate and enhance the sensory processing of the memoranda. In short, for the first time, we explored dynamic changes in fronto-parietal activity in childhood, its relationship with VSTM, and its impact upon subsequent sensory processing. The high variability in performance across a number of VSTM/WM tasks in childhood is typically accounted for in terms of immature maintenance abilities during childhood, and their protracted development. Here, we demonstrate that prestimulus oscillatory power changes can also be critical for subsequent “maintenance” success in childhood.

Low-frequency oscillations, such as those studied here, may play a particularly critical role in the regulation of cortical excitability in anticipation of stimuli (Schroeder and Lakatos 2009). One possibility is that these slow frequency modulations provide an underlying structure within which object-based representations, underpinned by higher frequency smaller local neuronal pools, can be organized (e.g., Axmacher et al. 2010). Of course, this need not occur just in anticipation of the memoranda, as we observe here, but these slow oscillations could also presumably be important during the lengthy VSTM maintenance delay, in anticipation of the probe stimulus (as in Axmacher et al. 2010).

Including the accuracy as well as the load contrast also provides an additional important control condition for our developmental data. Differences between high and low VSTM-load trials may depend on differences in basic visual processing across the 2 stimulus types, as well as differences in encoding. Considering the accuracy contrast in the Load-4 condition only is a good control in this context. Both trial types included in this contrast are perceptually identical, but in one case leading to failures of memory (incorrect trials) and in the other case to successes (correct trials). If a component network is modulated by subsequent accuracy, this can only result from an endogenous mechanism rather than a difference in sensory input.

Here, we demonstrate that the differences between subsequently correct and incorrect trials emerge prior to the onset of the memoranda. Interestingly, the same pattern of prestimulus relevance to later memory is not significantly present in the adult data, even though the network itself is very similar across the 2 groups. It is necessarily difficult to compare directly the data of children and adults. However, in this case, the performance of the 2 groups does not differ significantly, and the effect of interest occurs prior to the onset of the stimulus. These 2 features make it unlikely that this difference across the 2 groups stems from the slightly different paradigms, or from differences in task difficulty. Instead, one possibility is that the sources of variability in performance for our 2 groups are different. For children, anticipating accurately the onset of the memoranda may drive substantial variability in encoding and thus subsequent VSTM performance (Cowan et al. 2011). In contrast, the variability in performance for the adults could stem from alternative mechanisms later in the trial, or even at the point of recognition, which we are not able to capture here.

### Electrophysiological Measures of Functional Connectivity

To our knowledge, this is the first attempt to use electrophysiology to parcellate and study the variability in activation and connectivity of cortical networks in childhood, and it confers a number of advantages relative to more traditional measures of functional connectivity (e.g., fc-fMRI). Through its high sensitivity to changes in magnetic fields arising from electrical activity in the brain and fine-grained temporal resolution, MEG directly measures neural activity that is not complicated by metabolic or vasculature changes (e.g., Harris et al. 2011; Church et al. 2012). This has large benefits for interpreting differences between individuals and across developmental time. Furthermore, because we use the electrophysiological nature of these networks to explore the relationship with trial-to-trial successes/failures, it is possible to identify with much greater precision when in a trial the activity of a network is critically related to performance. This is vital information for assigned the functional role of that particular network. Secondly, this approach represents a substantial advance over previous methods of using EEG or MEG data to look at functional connectivity. Using source-projected MEG data, we explore connectivity at the level of cortex, rather than at the sensor level (e.g., Ortiz et al. 2012). Finally, the ICA approach we use is not provided with any spatial information about the networks. Network identification is entirely data-driven, arising from the temporal structure of the oscillations in those regions of cortex, without the need for any a priori assumptions.

### Future Directions

In the current study, we used relatively low-frequency oscillations, and our connectivity analyses were performed on the temporally down-sampled envelope of those oscillations. This approach has the advantage of minimizing the likelihood of identifying spurious connections (Brookes et al. 2011; Luckhoo et al. 2012). However, while the temporal precision of this approach is substantially better than that permitted by fc-fMRI approaches, we are still currently ignoring large amounts of high-frequency neural activity. In the future, a possible methodological advance would be to explore the role in maintenance for networks at other frequencies (e.g., Brookes et al. 2011), or to explore the relationship between low-frequency changes and high-frequency activity (e.g., Axmacher et al. 2010).

There are also a number of possible new applications of this approach. While here we explore the relationship between trial-to-trial variability in performance and dynamic fluctuations in fronto-parietal activity in childhood, a future application could explore variability in performance across children. One possibility is that the large differences in ability that exist across children can be partly explained by individual differences in functional connectivity, apparent both at rest and during task performance, in fronto-parietal connectivity. The combination of this approach with other types of neuroimaging, such as structural MRI and diffusion tensor imaging, could enable us to relate the protracted structural maturation of these networks (e.g., Giedd et al. 1999) to the extent to which the pattern of neural oscillations can be communicated from 1 node of the network to another. In short, we think that in the future this approach could complement other neuroimaging methodologies, and chart differences in dynamic neural activity across typical and atypical development.

## Funding

D.E.A. was supported by a British Academy Postdoctoral Fellowship and by the Medical Research Council (United Kingdom) intramural program (MC-A060-5PQ40). A.C.N. is supported by a Wellcome Trust Senior Investigator Award (104571/Z/14/Z). G.S. is supported by the James S. McDonnell Foundation Understanding Human Cognition Scholar Award. M.W.W. and A.C.N. are funded by an MRC/EPSRC UK MEG Partnership award (MR/K005464/1) and the National Institute for Health Research (NIHR) Oxford Biomedical Research Centre based at Oxford University Hospitals Trust Oxford University. Both D.E.A. and G.S. were also supported by a John Fell Oxford University Press grant. Funding to pay the Open Access publication charges for this article was provided by UK Medical Research Council (MC-A060-5PQ40).

## Notes

*Conflict of Interest*: None declared.
